# Quality of life of HIV-negative, previously healthy individuals following cryptococcal meningoencephalitis

**DOI:** 10.1038/s41598-021-83176-2

**Published:** 2021-02-11

**Authors:** Owen Dean, Seher Anjum, Terri Scott, Lillian Ham, Katherine Traino, Jing Wang, Sally Hunsberger, John H. Powers, Kieren A. Marr, Joseph Snow, Peter R. Williamson

**Affiliations:** 1 Laboratory of Clinical Immunology and Microbiology, National Institute of Allergy and Infectious Diseases, National Institutes of Health, Bld 10, Rm 11C208, 9000 Rockville Pike, Bethesda, MD 20892 USA; 2 National Institute of Mental Health, National Institutes of Health, Bethesda, MD USA; 3 Biostatistics Research Branch, National Institutes of Allergy and Infectious Diseases, National Institutes of Health, Bethesda, MD USA; 4 Clinical Research Directorate, Frederick National Laboratory for Cancer Research, Frederick, MD USA; 5 Johns Hopkins University School of Medicine, Baltimore, MD USA

**Keywords:** Medical research, Fungal infection, Quality of life

## Abstract

The morbidity and mortality of cryptococcal meningoencephalitis (CM) in previously healthy, HIV-negative individuals is increasingly recognized. We administered a healthcare associated quality of life (QOL) survey to the largest longitudinally followed cohort of these patients in the United States. We identified moderate or severe self-reported impairment in at least one QOL domain in 61% of subjects at least one year following diagnosis. Self-reported cognitive impairment was noted in 52% and sleep disturbance was noted in 55%. This is the first comprehensive study of cross-sectional long-term QOL in previously healthy patients following cryptococcal infection.

## Introduction

*Cryptococcus* is a cause of fatal meningoencephalitis that kills 250,000 people worldwide each year ^1^. Mortality of CM is high in both immunocompetent and immunocompromised populations, reaching 42% and 72% at one year respectively^2^. Between 20–70% of CM survivors are estimated to have long-term disability following infection, similar to other causes of acute infectious meningitis like tuberculosis, although the number and quality of current studies is limited^2^.

In the U.S., 17–30% of non-HIV infected individuals with CM have no apparent underlying condition, accounting for approximately 1000 cases per year ^1^. This group has some of the highest mortality rates in the U.S.^3^ and significant disease sequelae including a fronto-subcortical syndrome^4^, hearing loss^5^, vision loss, and spinal arachnoiditis^6^, associated with direct fungal damage as well as a post-infectious inflammatory response syndrome (PIIRS)^7^. The effect of these sequelae on quality of life (QOL) is not known. Here, we examined QOL of HIV-negative, previously healthy individuals at least one year following CM diagnosis using the patient-reported survey instrument Quality of Life in Neurological Disorders or Neuro-QoL.

## Results

Of 66 subjects eligible for survey participation, 56 (85%) completed the survey between January 2020 and June 2020. Ten surveyed subjects (18%) did not have CNS disease; 9 had isolated pulmonary disease and one had confirmed osteomyelitis of the thoracic vertebrae (T4-T5). The demographic and clinical characteristics of the surveyed population are shown in Table 1. Fifteen patients (33%) with CNS disease received corticosteroid therapy, with varied tapers and dosing, during their treatment course. The most common pre-existing conditions in surveyed subjects were idiopathic CD4 + lymphopenia (14%) and diabetes mellitus (7%). All known pre-existing conditions prior to diagnosis are listed in Supplemental Table 2.

**Table 1:** Patient demographic and clinical characteristics.

	CNS disease, n = 46	Non-CNS disease, n = 10
Age at Diagnosis, median years [IQR]	51.2 [37.2–60.4]	42.9 [34.0–53.3]
Diagnosis to HRQOL survey, median years [IQR]	5.4 [2.4–8.4]	8.3 [4.8–8.6]
Gender, male/female (% male)	27/19 (59)	3/7 (30)
Race, n (%)		
White	36 (78)	7 (70)
Black/African American	3 (7)	1 (10)
Asian	3 (7)	1 (10)
American Indian/Alaska Native	1 (2)	0 (0)
Multiracial or other	3 (7)	1 (10)
Ethnicity, n (%) Hispanic	4 (4)	1 (2)
Cryptococcal Isolate		
*C. neoformans*	16 (35)	3 (30)
*C. gattii*	12 (33)	2 (20)
Unknown	18 (39)	5 (50)
CSF glucose at diagnosis, median [IQR] mg/dL n = 22	30 [19–51]	N/A
CSF Cryptococcal Antigen titer at diagnosis, median [IQR] n = 22	1:1236 [1:448–1:2048]	N/A
Neurosurgical Intervention during hospitalization*, no. (%)	19 (44)	N/A

CSF: cerebrospinal fluid; *18/19 with neurosurgical intervention (95%) had a ventriculoperitoneal shunt placement, 1 (5%) had a lumbar drain placement.

**Figure 1:**
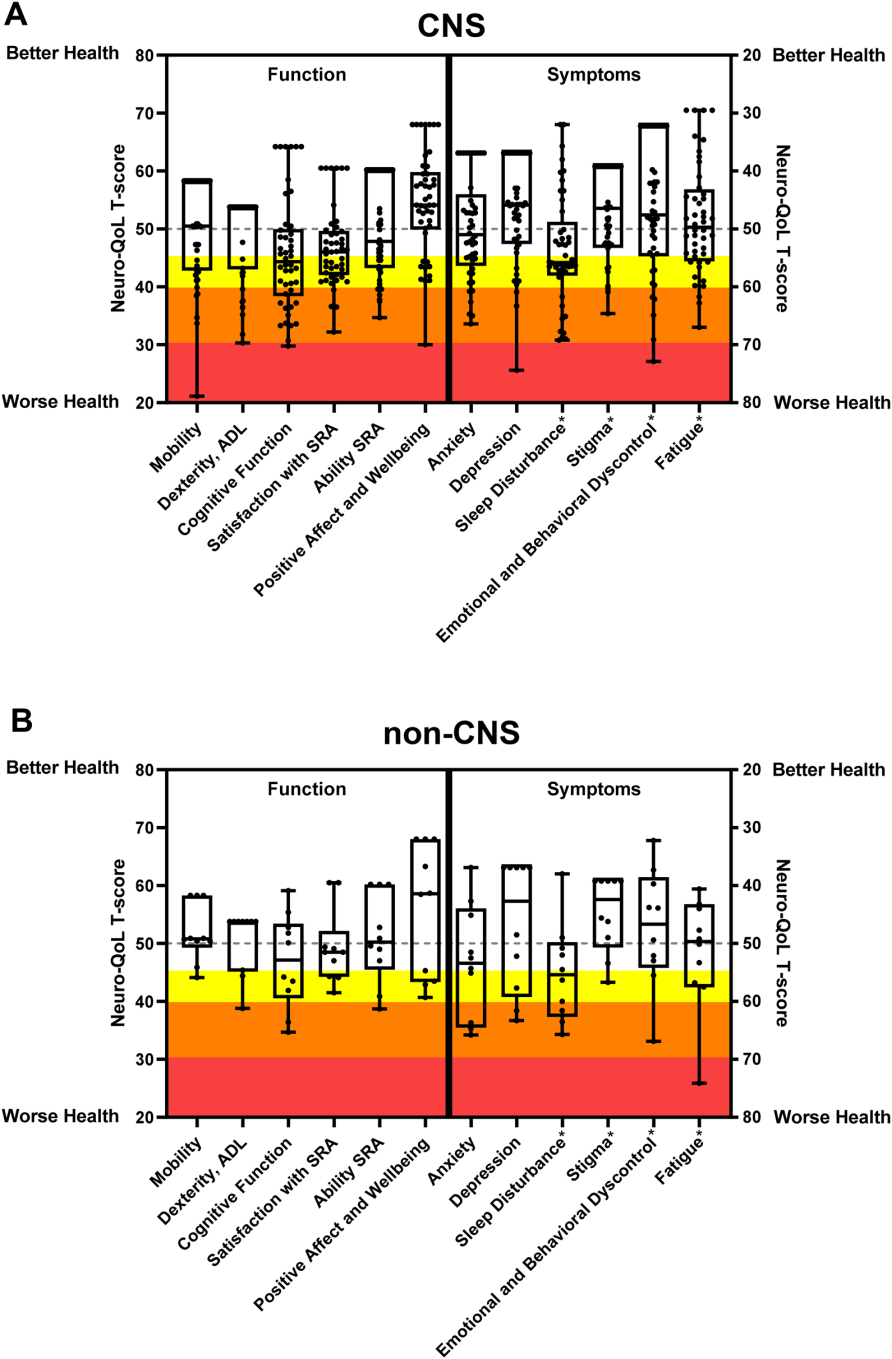
Patient reported quality of life following microbial recovery from cryptococcosis by population scaled Neuro-QoL domains. (**A**) subjects with CNS cryptococcosis (n = 46). (**B**) non-CNS cryptococcosis subjects (n = 10). Box plots show median, 25th, and 75th percentiles. The yellow region designates mild symptoms or impairment, orange, moderate, and red, severe. The gray dotted line represents the mean T-score (50) of the U.S. population reference for each Neuro-QoL domain. The asterisk* indicates measures that were centered to U.S. clinical reference population. All other domains were centered to a U.S. general population reference. Abbreviations: CNS, central nervous system, ADL, activities of daily living, SRA, social roles and activities.

The distributions of the population scaled Neuro-QoL T-scores with CM and non-CNS cryptococcosis are summarized in Fig. 1 and Supplemental Tables 3 & 4. Sixty-one percent of those with CNS disease and 50% in the non-CNS group had evidence of moderate or severe impairment in at least one QOL domain. The median total number of moderately and severely impaired QOL domains was equal in both groups, 1 (IQR [0–3]) in those with CNS disease and 1 (IQR [0–3]) in the non-CNS group .

In the CNS cryptococcosis group, sleep disturbance (55% with at least mild symptoms) and cognitive function (52% with at least mild impairment) were reported as the most affected domains (Supplemental Table 3). The domain with the lowest frequency of symptoms or impairment in the CNS group was stigma (16% with at least mild symptoms).

**Figure 2:**
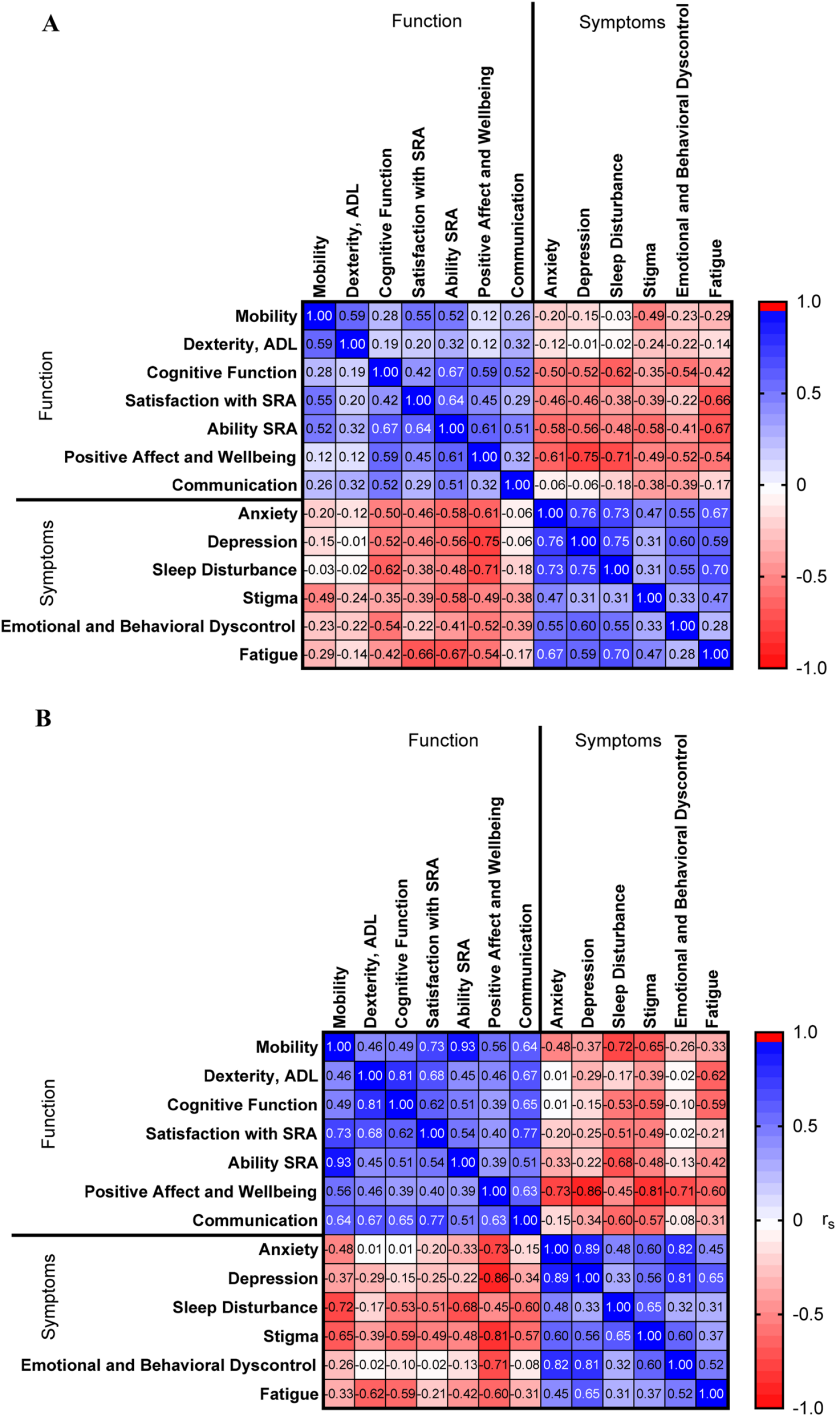
Correlation matrices of Neuro-QoL Domain T-scores for (**A**) CNS subjects (n = 36) and (**B**) non-CNS subjects (n = 10). r_s_ = Spearman correlation coefficient.

In the CNS cryptococcosis group, sleep disturbance T-scores were positively correlated with fatigue (r_s_ = 0.70, *P* < 0.0001), anxiety (r_s_ = 0.73, *P* < 0.0001), depression (r_s_ = 0.75, *P* < 0.0001), as well as negatively correlated with positive affect and wellbeing (r_s_ = -0.71, *P* < 0.0001). Depression T-scores were positively correlated with anxiety (r_s_ = 0.76, *P* < 0.0001) and negatively correlated with positive affect and wellbeing (r_s_ = -0.75, *P* < 0.0001). All correlations between Neuro-QoL T-score domains are shown in Fig. 2.

Within the non-CNS group, mild symptoms were noted by at least one subject in all reported QOL domains. Similar to the CNS group, cognitive function (50% with at least mild impairment) and sleep disturbance (50% with at least mild symptoms) were the most reported affected domains. The domains with the lowest frequency of reported symptoms or impairment were mobility, communication, and stigma at 10%, respectively (Supplemental Table 4).

The presence of a neurosurgical intervention in the CNS group during hospital course was not associated with a difference in the median number of moderately or severely self-reported impaired domains (1.0 versus 1.0, *P* = 0.338).

Self-reported cognitive function T-scores were not significantly associated with diagnostic CSF cryptococcal antigen (coefficient = 24.79, *P* = 0.2834) or CSF glucose (coefficient = 0.7921, *P* = 0.1137). CSF:blood glucose ratios at 100 + /- 50 days past diagnosis were not significantly associated with cognitive function T-scores (coefficient = -0.117, *P* = 0.991). Longitudinal plots of CSF:blood glucose ratios and CSF white blood cell count are shown in Supplementary Fig. 1. Notable are the traces of some patients maintaining low glucose ratios for weeks and months following diagnosis and perceived infection control and a qualitative note that some these traces were associated with low cognitive function T-scores.

Among the overlapping CNS group patients who underwent comprehensive neuropsychological (NP) testing, self-reported cognitive function T-score was positively correlated with global NP T-score (r_s_ = 0.552, *P* = 0.067, n = 12), with declines in function in both. In the same way, Beck Depression Inventory (BDI) scores were positively correlated with the depression T-scores (r_s_ = 0.582, *P* = 0.05) and Beck Anxiety Inventory (BAI) scores were positively correlated with anxiety T-scores (r_s_ = 0.605, *P* = 0.04).

## Discussion

In this study we described the patient-reported QOL of one of the largest cohort of previously healthy patients with cryptococcosis. We found that the majority of subjects in the NIH cohort endorsed significant QOL issues in at least one domain, with cognitive function and sleep disturbance being the most commonly self-reported affected domains. Impairment in mobility, fine motor, ability to participate in social roles and activities (SRA), and satisfaction with SRA domains was also identified; these four Neuro-QoL domains correlate with common measures of activities of daily living (ADL) and instrumental ADL completion (Barthel and Lawton IADL Indices, respectively^8,9^.

The identification of self-reported cognitive impairment in 52% of subjects with CNS disease aligns with previous reports of neurocognitive deficits^10–12^. Our group recently defined a persistent fronto-subcortical syndrome in HIV-negative subjects with CM^4^, several of the same patients being involved in this study. Although interpretation is limited by the small number of overlapping subjects (n = 12), summative NP T-score trended towards a positive correlation with self-reported cognitive function T-score. Given the varied distribution of responses to the cognitive function Neuro-QoL measure and the lack of a clear ceiling effect in the data (Supplementary Fig. 1A), this measure may be a useful adjunct and a partial alternative when it is too costly or time consuming to conduct intensive formal NP testing for this disease in outpatient clinic settings. Further study is necessary to validate this finding, given that self-reported cognitive complaints often do not correlate with objective cognitive function and may be more strongly correlated with emotional distress^13–16^.

The identification of sleep disturbance in 56% of subjects with CNS disease was interesting. Given the low frequency of pre-existing sleep conditions in the CNS group (3 subjects with sleep apnea), we believe it is likely that cryptococcal infection may be driving this symptomatology. Although self-reported impaired sleep is known to be a long-term consequence of viral, bacterial^17^, and tuberculous meningitis^18^, cryptococcal meningitis has not been previously associated with a change in sleep quality.

The anxious and depressive symptoms identified in 30% and 17% of subjects with CNS disease respectively (Supplemental Table 3) corresponds with our groups’ previous report as well. In Traino et al. 2019, the median self-reported anxiety score using the BAI was 5 in previously healthy subjects with CNS cryptococcosis. Subjects with CNS cryptococcosis self-reported BAI scores an average of 10.5 and BDI scores6.0 higher than a control group of subjects with mild cognitive impairment^19^. We identified that BAI and BDI scores were moderately correlated with their respective self-reported QOL domain scores in the 12 subjects with overlapping data. Interestingly, sleep disturbance may be linked with the prevalence of anxiety and depression in this population; given the highly correlated QOL scores on these metrics (Fig. 2A).

We also identified significant self-reported impairment in many QOL domains in the non-CNS cryptococcosis group. Despite being limited by a small sample size, it seems there is significant impairment present in several QOL domains including cognitive function and sleep disturbance, similar to the CNS group (Fig. 1b). We had expected this group to be less disabled given the lack of CNS involvement. Marr et al. 2020, who surveyed a cohort of HIV-negative cryptococcosis patients, found that HIV-negative patients with non-CNS cryptococcosis had a higher aggregate QOL score, the RAND-36 total score, than those with CNS disease at diagnosis ^20^. We can think of several explanations for these data, that the genetic or acquired defects that predispose this population to cryptococcal infection may also chronically affect other body systems, the inflammatory response to cryptococcosis in the lungs is also a source of debilitation, and/or these are neuropsychological sequalae common to the diagnosis and treatment of life-threatening illness.

Antifungal and/or immunosuppressive therapy may also contribute to QOL impairment in cryptococcosis. Amphotericin B treatment, both with intrathecal and intravenous dosing, has been associated with confusion and disorientation hours to weeks after initiation, with symptoms typically resolving after treatment cessation^21^. Corticosteroid therapy has also been associated with mood disturbance, hypomania, psychosis, depression, and cognitive dysfunction. While typically considered dose-dependent and self-resolving, corticosteroids may also cause persistent neuropsychological dysfunction^22–24^. Variance in treatment courses precluded treatment subgroup analyzes in this cohort.

This study is primarily limited by its cross-sectional design. Given the varied time points, disease courses, and treatment courses, it was not possible to identify predictive laboratory or clinical measures of future QOL impairment. Further complicating the interpretation of these data is the varied past medical history of this patient cohort, while although likely not as confounding as the biopsychosocial complexities present in an HIV-positive cohort with cryptococcosis, these conditions may be altering the QOL domain results in this study. The extended median time from diagnosis of the QOL survey of greater than 5 years may increase the chances of alternative diagnoses confounding our results. This extended time period is likely secondary to the many years this cohort has been followed at the NIH Clinical Center and that many subjects were referred to the NIH on an outpatient basis months or years following their initial diagnosis at outside institutions. We also note that the patients referred to the NIH Clinical Center may be of higher acuity than the HIV-negative patients routinely seen in other U.S. hospital systems, because many are referred due to refractivity in clinical response.

We did not formally assess internal consistency or test–retest reliability of Neuro-QoL measures, given the relatively large number of number of survey items and our small cohort size. We pragmatically chose Neuro-QoL measures because of their previous validation in several neurological diseases with varied sequalae^8,25–30^; making it less likely that survey characteristics would be significantly affected by unique aspects of the HIV-negative cryptococcosis patient cohort. Larger multi-site and multinational studies incorporating patient and caregiver interviewers are required to formally validate Neuro-QoL measures in this rare disease population^31^. The findings of this descriptive study emphasize that cryptococcal meningoencephalitis in the previously healthy, HIV-negative population is associated with chronic sequalae that impact patient QOL years following infection. Following recovery from infection, despite an apparent successful clinical course, care givers should pay particular attention to patient cognitive function and sleep changes. In addition, these results demonstrate the utility of a patient-focused long-term outcome vehicle to demonstrate and compare experimental treatment modalities. Future prospective studies utilizing patient-reported QOL measures like the Neuro-QoL question battery may thus help to better understand the sequalae of this severe disease and identify predictive biomarkers and therapeutics to improve patient outcomes.

## Methods

### Study population

All previously healthy patients > 18 years old with a confirmed diagnosis of cryptococcosis by histopathology, culture, or cryptococcal antigen evaluated at the NIH Clinical Center from January 2013-January 2019 were invited to participate in this study and were recruited from an ongoing National Institute of Allergy and Infectious Disease study, *Cryptococcosis in Previously Healthy Adults* (NCT00001352). All patients had been seen at the National Institutes of Health Clinical Center, Bethesda, Maryland, and informed consent was obtained. All experimental protocols were approved by the research ethics committee and Institutional Review Board of the NIAID (NIH). All methods were carried out in accordance with relevant guidelines and regulations. Exclusion criteria included being within 1 year of cryptococcal diagnosis, HIV seropositivity, chemotherapeutic agent use, underlying malignancy, and/or monoclonal antibody use prior to cryptococcal diagnosis. Patients received standard antifungal therapy after diagnosis, at least one course of amphotericin B followed by prolonged maintenance therapy with fluconazole. A minority of patients also received immunosuppressive therapy during their hospitalization. Patients were consented and mailed a survey packet to complete at home. All methodology was approved by the National Institute of Allergy and Infectious Disease Intramural Institutional Review Board.

### Survey and clinical data collection

The Quality of Life in Neurological Disorders (Neuro-QoL) is a patient-reported measurement system that evaluates and monitors the physical, mental, and social effects experienced by adults and children living with neurological conditions^8^. These questions have been validated, and shown adequate internal consistency and re-test reliability, in patients with other conditions that confer chronic neurologic morbidity, such as epilepsy, multiple sclerosis, Parkinson Disease, and stroke. We utilized a paper survey battery consisting of 13 Adult Neuro-QoL short-forms assessing 7 functional and 6 symptomatic domains in English (Supplemental Table 1). Some participants with visual difficulties had assistance from their caregivers in completing the forms. Caregivers did not interpret or reply to questions. Results were collected over a 6-month period.

Surveys were scored via HealthMeasures Scoring Service (https://www.assessmentcenter.net/ac_scoringservice). Reported T-scores have a mean of 50 and a standard deviation (SD) of 10 centered and calibrated to a general or clinical United States population (Supplemental Table 1). For functional measures, higher scores are representative of better health. For symptom measures, higher scores are representative of worse health. Following convention, scores 0.5–1.0 SD worse than the reference mean were considered mild symptoms or impairment, 1.0–2.0 SD or worse, moderate impairment, and 2.0 SD or more, severe impairment^8^.

Descriptive data from participants was collected through the Biomedical Translational Research Information System (http://btris.nih.gov). Comprehensive neuropsychological (NP) evaluation data, as reported in Traino et al. 2019, was used to compare functional measures with self-reported QOL domain T-scores. Briefly, NP evaluation assessed psychomotor, information processing, executive function, learning, memory, language, attention, and visuospatial function, summarized in a demographically-corrected global T-score. Mood symptoms were assessed with the Beck Depression Inventory II (BDI) and the Beck Anxiety Inventory (BAI)^4^^.^

### Statistics

Continuous data were compared with two-tailed, non-paired Mann–Whitney U tests and frequency data with chi-square test. Associations between T-scores and clinical continuous metrics were calculated with simple linear regressions. Significance was considered alpha < 0.05. Spearman’s rank correlation coefficient was calculated between individual domain T-scores and NP test scores. Although statistical comparisons between the CNS and non-CNS groups were not performed given the small samples sizes in the latter group, the groups were displayed separately given the notable differences in clinical presentation, treatment, and sequalae between the populations.

## Supplementary Information

Supplementary Information.

## References

[1] Williamson PR (2017). Cryptococcal meningitis: epidemiology, immunology, diagnosis and therapy. Nat. Rev. Neurol..

[2] Pasquier E (2018). Long-term mortality and disability in cryptococcal meningitis: a systematic literature review. Clin. Infect. Dis.: Off. Publ. Infect. Dis. Soc. Am..

[3] Brizendine KD, Baddley JW, Pappas PG (2013). Predictors of mortality and differences in clinical features among patients with cryptococcosis according to immune status. PLoS ONE.

[4] Traino K (2019). HIV-negative cryptococcal meningoencephalitis results in a persistent frontal-subcortical syndrome. Sci. Rep..

[5] King KA (2019). Audiologic and otologic complications of cryptococcal meningoencephalitis in non-HIV previously healthy patients. Otol. Neurotol..

[6] Panackal AA (2017). Spinal arachnoiditis as a complication of cryptococcal meningoencephalitis in non-HIV previously healthy adults. Clin. Infect. Dis.: Off. Publ. Infect. Dis. Soc. Am..

[7] Neal LM (2017). CD4(+) T cells orchestrate lethal immune pathology despite fungal clearance during cryptococcus neoformans meningoencephalitis. MBio.

[8] Cella D (2012). Neuro-QOL: brief measures of health-related quality of life for clinical research in neurology. Neurology.

[9] 9Neuro-QoL Technical Report: Development and Initial Validation of Patient-Reported Item Banks for use in Neurological Research and Practice (2015).

[10] Chen CH (2012). Neuro-psychological sequelae in HIV-negative cryptococcal meningitis after complete anti-fungal treatment. Acta Neurol. Taiwan.

[11] Chen MH (2015). Long-term neuropsychological sequelae in HIV-seronegative cryptococcal meningoencephalitis patients with and without ventriculoperitoneal shunts: a cine MRI study. Behav. Neurol..

[12] Lu CH (2011). Assessing the chronic neuropsychologic sequelae of human immunodeficiency virus-negative cryptococcal meningitis by using diffusion tensor imaging. AJNR Am. J. Neuroradiol..

[13] Hill NL (2016). Subjective cognitive impairment and affective symptoms: a systematic review. Gerontologist.

[14] Yoo-Jeong M (2018). Associations of mood on objective and subjective cognitive complaints in persons living with HIV/AIDS. J. HIV AIDS.

[15] Pranckeviciene A, Deltuva VP, Tamasauskas A, Bunevicius A (2017). Association between psychological distress, subjective cognitive complaints and objective neuropsychological functioning in brain tumor patients. Clin. Neurol. Neurosurg..

[16] Burmester B, Leathem J, Merrick P (2016). Subjective cognitive complaints and objective cognitive function in aging: a systematic review and meta-analysis of recent cross-sectional findings. Neuropsychol. Rev..

[17] Schmidt H (2006). Sleep disorders are long-term sequelae of both bacterial and viral meningitis. J. Neurol. Neurosurg. Psychiatry.

[18] Pardasani V, Shukla G, Singh S, Goyal V, Behari M (2008). Abnormal sleep-wake cycles in patients with tuberculous meningitis: a case-control study. J. Neurol. Sci..

[19] Bartizal K (1992). In vitro antifungal activities and in vivo efficacies of 1,3-beta-D-glucan synthesis inhibitors L-671,329, L-646,991, tetrahydroechinocandin B, and L-687,781, a papulacandin. Antimicrob. Agents Chemother..

[20] Marr KA (2019). A multicenter, longitudinal cohort study of cryptococcosis in HIV-negative people in the United States. Clin. Infect. Dis.: Off. Publ. Infect. Dis. Soc. Am..

[21] Warstler A, Bean J (2016). Antimicrobial-induced cognitive side effects. Ment. Health Clin..

[22] Brown ES, Chandler PA (2001). Mood and cognitive changes during systemic corticosteroid therapy. Prim. Care Companion J. Clin. Psychiatry.

[23] Judd LL (2014). Adverse consequences of glucocorticoid medication: psychological, cognitive, and behavioral effects. Am. J. Psychiatry.

[24] Savas M (2020). Systemic and local corticosteroid use is associated with reduced executive cognition, and mood and anxiety disorders. Neuroendocrinology.

[25] Fox RS, Peipert JD, Vera-Llonch M, Phillips G, Cella D (2020). PROMIS(R) and Neuro-QoL(TM) measures are valid measures of health-related quality of life among patients with familial chylomicronemia syndrome. Expert Rev. Cardiovasc. Ther..

[26] Carlozzi NE (2020). Responsiveness to change over time and test-retest reliability of the PROMIS and Neuro-QoL mental health measures in persons with Huntington disease (HD). Qual. Life Res..

[27] Bode RK (2010). Development and validation of participation and positive psychologic function measures for stroke survivors. Arch. Phys. Med. Rehabil..

[28] Lai JS (2015). Validation of the Neuro-QoL measurement system in children with epilepsy. Epilepsy Behav..

[29] Nowinski CJ (2016). Neuro-QoL health-related quality of life measurement system: validation in Parkinson's disease. Mov. Disord..

[30] Victorson D (2015). Measuring resilience after spinal cord injury: Development, validation and psychometric characteristics of the SCI-QOL Resilience item bank and short form. J. Spinal Cord Med..

[31] Slade A (2018). Patient reported outcome measures in rare diseases: a narrative review. Orphanet. J. Rare Dis..

