# Supplementary material for: Quality of life of HIV-negative, previously healthy individuals following cryptococcal meningoencephalitis
Source: Sci Rep. 2021 Feb 11;11:3673. doi: 10.1038/s41598-021-83176-2 (PMC7878796; doi:10.1038/s41598-021-83176-2)
Supplement: Supplementary file 1 — Supplementary Information. [file 41598_2021_83176_MOESM1_ESM.pdf]

Quality of life of HIV-negative, previously healthy individuals following cryptococcal meningoencephalitis.

Owen Dean[1], Seher Anjum[1], Terri Scott[1], Lillian Ham[2], Katherine Traino[2], Jing Wang[4], Sally Hunsberger[3], John H Powers III[4], Kieren A. Marr[5], Joseph Snow[2], Peter R Williamson[1]

[1]Laboratory of Clinical Immunology and Microbiology, National Institute of Allergy and Infectious Diseases, National Institutes of Health, Bethesda, MD USA

[2]National Institute of Mental Health, National Institutes of Health, Bethesda, MD USA

[3]Biostatistics Research Branch, National Institutes of Allergy and Infectious Diseases, National Institutes of Health, Bethesda, MD USA

[4]Clinical Research Directorate, Frederick National Laboratory for Cancer Research, Frederick, MD USA

5Johns Hopkins University School of Medicine, Baltimore, MD USA

Corresponding author:

Peter R. Williamson

Bld 10, Rm 11C208

9000 Rockville Pike

Bethesda, MD 20892

(301) 443-8339

williamsonpr@mail.nih.gov

Running Title: QOL after HIV-negative cryptococcosis

Supplemental Table 1: **Adult Neuro-QoL Short Forms Surveys**

**Domain Name**	**Reference Centering Population***	**Edits to Survey**
Neuro-QOL Item Bank v1.0- Ability to Participate in Social Roles and Activities	General U.S. Population Wave 1b	N/A
Neuro-QOL Item Bank v1.0- Fatigue	Clinical population Wave 1a	N/A
Neuro-QOL Item Bank v1.0- Positive Affect and Well-Being	General U.S. Population Wave 1b	N/A
Neuro-QOL Item Bank v2.0- Cognition Function	General U.S. Population Wave 1b	N/A
Neuro-QOL Item Bank v1.0- Stigma	Clinical population Wave 1a	N/A
Neuro-QOL Item Bank v1.0- Lower Extremity Function (Mobility)	General U.S. Population Wave 1b	N/A
Neuro-QOL Item Bank v1.0- Sleep Disturbance	Clinical population Wave 1a	N/A
Neuro-QOL Item Bank v1.1- Satisfaction with Social Roles and Activities	General U.S. Population Wave 1b	N/A
Neuro-QOL Item Bank v1.0- Anxiety	General U.S. Population Wave 1b	N/A
Neuro-QOL Item Bank v1.0- Depression	General U.S. Population Wave 1b	Question EDDEP39 "I felt I had no reason for living" was removed.
Neuro-QOL Item Bank v1.0- Emotional and Behavioral Dyscontrol	Clinical population Wave 1a	N/A
Neuro-QOL Item Bank v1.0- Upper Extremity Function (Fine Motor, ADL)	General U.S. Population Wave 1b	N/A
Neuro-QoL Item Bank v1.0- Communication	Not-scaled	N/A

***The Neuro-QoL clinical reference population consisted of 553 English-speaking patients: 209 with history of stroke, 183 with epilepsy, 84 with MS, 59 with Parkinsons disease, and 18 with amyotrophic lateral sclerosis.**

Supplemental Table 2: **Pre-existing Conditions**

	CNS disease, n=46	Non-CNS, n=10
Asthma	2 (4%)	1 (10%)
Diabetes mellitus	3 (7%)	1 (10%)
Heart Failure	1 (2%)	0
Hypothyroidism	2 (4%)	1 (10%)
Sleep apnea	3 (7%)	1 (10%)
Insomnia	1 (2%)	0
Sarcoidosis	4 (9%)	0
Multiple sclerosis	1 (2%)	0
Idiopathic CD4+ lymphopenia	7 (15%)	1 (10%)
Pulmonary Alveolar Proteinosis	2 (4%)	0
Dermatomyositis	1 (2%)	0
Recurrent ear infections	2 (4%)	0
Neoplasm**	3 (7%)	1 (10%)
Meniere's Disease	0	1 (10%)
Other Sensorineural Hearing Loss	2 (4%)	0
Anxiety	2 (4%)	1 (10%)
Depression	2 (4%)	1 (10%)
Opioid Abuse	1 (2%)	0
Seizure disorder	1 (2%)	0

*Type I: 1, Type II: 2

**CM: sarcoma, melanoma, squamous cell carcinoma/prostate cancer; non-CNS: basal cell carcinoma of the tongue

Supplemental Table 3: **Number and percentage of impaired CNS subjects (n=46) by Neuro-QoL domain**

		Impairment, n (%)			
	**Neuro-QoL Domain**	None	Mild	Moderate	Severe
Function	Mobility	**32 (70)**	**8 (17)**	**5 (11)**	**1 (2)**
	Dexterity, ADL	**33 (72)**	**5 (11)**	**8 (17)**	**0**
	Cognitive Function	**22 (48)**	**11 (24)**	**12 (26)**	**1 (2)**
	Satisfaction with SRA	**27 (59)**	**17 (27)**	**4 (9)**	**0**
	Ability to participate in SRA	**31 (67)**	**8 (17)**	**7 (15)**	**0**
	Positive Affect and Wellbeing	**36 (78)**	**9 (20)**	**0**	**1 (2)**
	Communication	**38 (83)**	**3 (7)**	**3 (7)**	**2 (4)**
Symptomatic	Anxiety	**32 (70)**	**8 (17)**	**6 (13)**	**0**
	Depression	**38 (83)**	**5 (11)**	**2 (4)**	**1 (2)**
	Sleep Disturbance	**21 (46)**	**16 (35)**	**9 (20)**	**0**
	Stigma	**39 (85)**	**4 (9)**	**3 (7)**	**0**
	Emotional and Behavioral Dyscontrol	**37 (80)**	**4 (9)**	**4 (9)**	**1 (2)**
	Fatigue	**35 (76)**	**8 (17)**	**3 (7)**	**0**

ADL: activities of daily living; SRA: social roles and activities

Supplemental Table 4: **Number and percentage of impaired non-CNS subjects (n=10) by Neuro-QoL domain**

		**Impairment, n (%)**			
	**Neuro-QoL Domain**	None	Mild	Moderate	Severe
Function	Mobility	**9 (90)**	**1 (10)**	**0**	**0**
	Dexterity, ADL	**8(80)**	**1 (10)**	**1(10)**	**0**
	Cognitive Function	**5(50)**	**3 (30)**	**2(20)**	**0**
	Satisfaction with SRA	**7 (70)**	**3(30)**	**0**	**0**
	Ability to participate in SRA	**8(80)**	**1(10)**	**1(10)**	**0**
	Positive Affect and Wellbeing	**7(70)**	**3(30)**	**0**	**0**
	Communication	**9 (90)**	**1(10)**	**0**	**0**
Symptomatic	Anxiety	**6 (60)**	**1(10)**	**3(30)**	**0**
	Depression	**7(70)**	**1(10)**	**2(20)**	**0**
	Sleep Disturbance	**6 (60)**	**2 (20)**	**2(20)**	**0**
	Stigma	**9(90)**	**1(10)**	**0**	**0**
	Emotional and Behavioral Dyscontrol	**9(90)**	**1(10)**	**1(10)**	**0**
	Fatigue	**7(70)**	**2 (20)**	**0**	**1(10)**

ADL: activities of daily living; SRA: social roles and activities

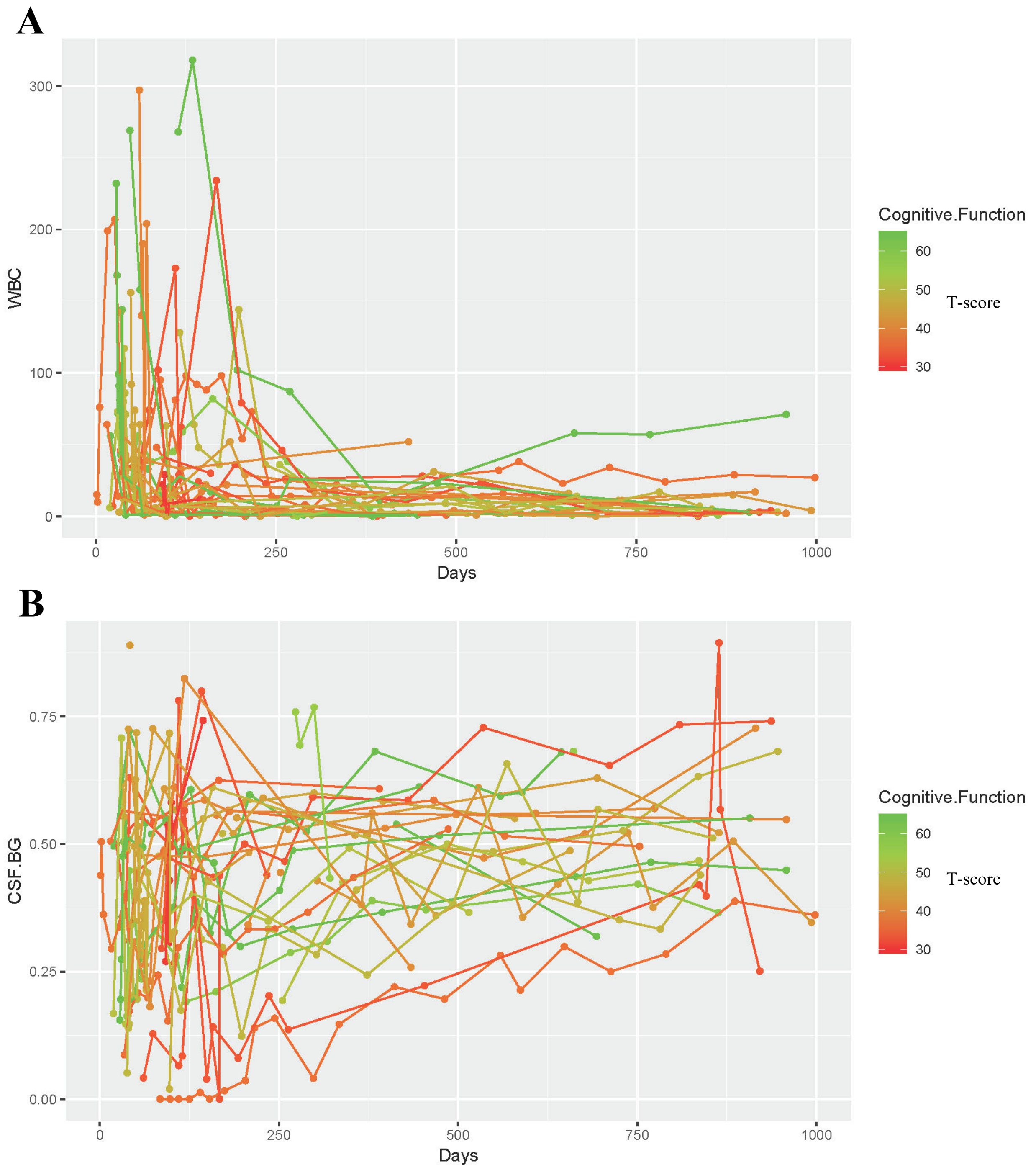
A
300
200
100
0
WBC
0
250
500
750
1000
Days
Cognitive.Function
60
50
40
30
T-score
B
0.75
0.50
0.25
0.00
CSF.BG
0
250
500
750
1000
Days
Cognitive.Function
60
50
40
30
T-score

Supplemental Figure 1: **Longitudinal cerebrospinal fluid (CSF) profiles of CNS cryptococcosis subjects with overlaid long-term Neuro-QoL cognitive function T-score (n=43).** A: CSF white blood cell (WBC) count versus days past diagnosis. B: CSF glucose:blood glucose (BG) ratio versus days past diagnosis. Individual subject data points are connected by lines. Subject data points and lines are colored by the Neuro-QoL cognitive function T-score with green being better scores, red being worse. All data points are representative of lumbar punctures (LP) performed at the NIH Clinical Center. All Neuro-QoL data was surveyed after the representative LP points shown on the diagram. Not all subjects had the same number of LPs performed. The timing of LPs was based on clinical condition and necessity.
